# 2-(*o*-Tol­yloxy)pyrimidine

**DOI:** 10.1107/S1600536809026609

**Published:** 2009-07-18

**Authors:** Nasir Shah Bakhtiar, Zanariah Abdullah, Seik Weng Ng

**Affiliations:** a Department of Chemistry, University of Malaya, 50603 Kuala Lumpur, Malaysia

## Abstract

The title compound, C_11_H_10_N_2_O, crystallizes with two mol­ecules in the asymmetric unit. The angle at the ether O atom is widened to 118.13 (15)° [117.89 (16)° for the second mol­ecule in the asymmetric unit]; the six-membered rings subtend a dihedral angle of 84.3 (1)° [87.4 (1)° in the second mol­ecule].

## Related literature

For 2-phenoxy­pyrimidine, see: Shah Bakhtiar *et al.* (2009 ▶).
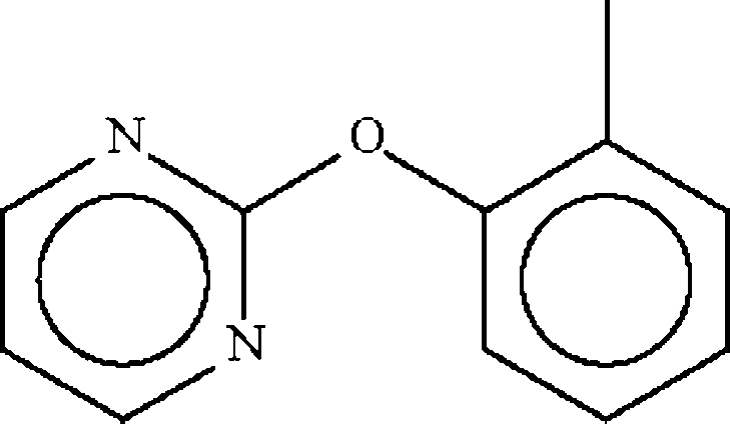


## Experimental

### 

#### Crystal data

C_11_H_10_N_2_O
*M* *_r_* = 186.21
Orthorhombic, 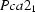

*a* = 7.5197 (2) Å
*b* = 12.7997 (3) Å
*c* = 20.3238 (4) Å
*V* = 1956.16 (8) Å^3^
*Z* = 8
Mo *K*α radiation
μ = 0.08 mm^−1^
*T* = 153 K
0.35 × 0.25 × 0.15 mm

#### Data collection

Bruker SMART APEX diffractometer
Absorption correction: none
12792 measured reflections
2317 independent reflections
2030 reflections with *I* > 2σ(*I*)
*R*_int_ = 0.028

#### Refinement

*R*[*F* ^2^ > 2σ(*F* ^2^)] = 0.033
*wR*(*F* ^2^) = 0.088
*S* = 1.02
2317 reflections
255 parameters
1 restraint
H-atom parameters constrained
Δρ_max_ = 0.16 e Å^−3^
Δρ_min_ = −0.19 e Å^−3^

### 

Data collection: *APEX2* (Bruker, 2008 ▶); cell refinement: *SAINT* (Bruker, 2008 ▶); data reduction: *SAINT*; program(s) used to solve structure: *SHELXS97* (Sheldrick, 2008 ▶); program(s) used to refine structure: *SHELXL97* (Sheldrick, 2008 ▶); molecular graphics: *X-SEED* (Barbour, 2001 ▶); software used to prepare material for publication: *publCIF* (Westrip, 2009 ▶).

## Supplementary Material

Crystal structure: contains datablocks global, I. DOI: 10.1107/S1600536809026609/bt2995sup1.cif

Structure factors: contains datablocks I. DOI: 10.1107/S1600536809026609/bt2995Isup2.hkl

Additional supplementary materials:  crystallographic information; 3D view; checkCIF report

## Figures and Tables

**Fig. 1.**
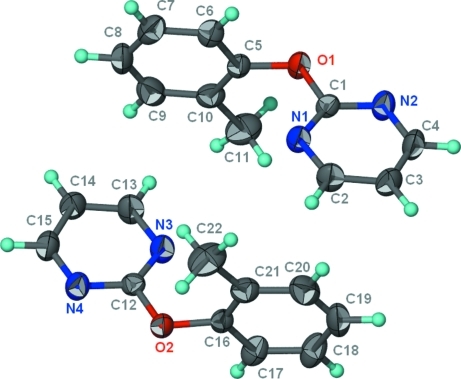
Anisotropic displacement ellipsoid plot (Barbour, 2001) of C11H10N2O at the 70% probability level; hydrogen atoms are drawn as spheres of arbitrary radius.

## supplementary crystallographic information

### Experimental

*o*-Cresol (2.16 g, 20 mmol) and sodium hydroxide (0.80 g, 20 mmol) were dissolved in water (50 ml) and to the solution was added 2-chloropyridimidine (2.30 g, 20 mmol) dissolved in THF (50 ml). The mixture was heated for 4 h. Water was added and the organic phase extracted with chloroform. The chloroform solution was dried over sodium sulfate; slow evaporation led to the formation of colorless crystals.

### Refinement

Carbon-bound H-atoms were placed in calculated positions (C—H 0.95–0.98 Å) and were included in the refinement in the riding model approximation, with *U*(H) set to 1.2–1.5*U*(C).

In the absence of anomalous scatterers, 2119 Friedel pairs were merged. The absolute structure was arbitrarily set.

### Crystal data

**Table:**

C_11_H_10_N_2_O	*F*(000) = 784
*M**_r_* = 186.21	*D*_x_ = 1.265 Mg m^−^^3^
Orthorhombic, *P**c**a*2_1_	Mo *K*α radiation, λ = 0.71073 Å
Hall symbol: P 2c -2ac	Cell parameters from 3906 reflections
*a* = 7.5197 (2) Å	θ = 2.6–27.9°
*b* = 12.7997 (3) Å	µ = 0.08 mm^−^^1^
*c* = 20.3238 (4) Å	*T* = 153 K
*V* = 1956.16 (8) Å^3^	Block, colorless
*Z* = 8	0.35 × 0.25 × 0.15 mm

### Data collection

**Table:**

Bruker SMART APEX diffractometer	2030 reflections with *I* > 2σ(*I*)
Radiation source: fine-focus sealed tube	*R*_int_ = 0.028
graphite	θ_max_ = 27.5°, θ_min_ = 1.6°
ω scans	*h* = −9→9
12792 measured reflections	*k* = −16→16
2317 independent reflections	*l* = −26→26

### Refinement

**Table:**

Refinement on *F*^2^	Primary atom site location: structure-invariant direct methods
Least-squares matrix: full	Secondary atom site location: difference Fourier map
*R*[*F*^2^ > 2σ(*F*^2^)] = 0.033	Hydrogen site location: inferred from neighbouring sites
*wR*(*F*^2^) = 0.088	H-atom parameters constrained
*S* = 1.02	*w* = 1/[σ^2^(*F*_o_^2^) + (0.0474*P*)^2^ + 0.3519*P*] where *P* = (*F*_o_^2^ + 2*F*_c_^2^)/3
2317 reflections	(Δ/σ)_max_ = 0.001
255 parameters	Δρ_max_ = 0.16 e Å^−^^3^
1 restraint	Δρ_min_ = −0.19 e Å^−^^3^

### Fractional atomic coordinates and isotropic or equivalent isotropic displacement parameters (Å^2^)

**Table:**

	*x*	*y*	*z*	*U*_iso_*/*U*_eq_	
O1	0.09137 (18)	0.69839 (11)	0.50102 (8)	0.0294 (3)	
O2	0.3463 (2)	0.79763 (11)	0.83583 (8)	0.0288 (3)	
N1	0.3704 (2)	0.76815 (13)	0.51731 (9)	0.0290 (4)	
N2	0.3222 (2)	0.59497 (13)	0.47797 (10)	0.0346 (4)	
N3	0.0668 (3)	0.73277 (14)	0.81493 (10)	0.0311 (4)	
N4	0.1165 (3)	0.90210 (14)	0.85951 (10)	0.0342 (4)	
C1	0.2716 (3)	0.68835 (14)	0.49926 (10)	0.0240 (4)	
C2	0.5457 (3)	0.75182 (17)	0.51316 (13)	0.0346 (5)	
H2	0.6241	0.8068	0.5252	0.041*	
C3	0.6173 (3)	0.65849 (18)	0.49217 (13)	0.0369 (5)	
H3	0.7421	0.6478	0.4895	0.044*	
C4	0.4976 (3)	0.58168 (19)	0.47530 (14)	0.0393 (6)	
H4	0.5424	0.5160	0.4611	0.047*	
C5	0.0193 (3)	0.78821 (16)	0.53078 (11)	0.0260 (4)	
C6	−0.0168 (3)	0.87417 (16)	0.49211 (12)	0.0302 (5)	
H6	0.0168	0.8751	0.4471	0.036*	
C7	−0.1029 (3)	0.95922 (17)	0.51989 (13)	0.0369 (5)	
H7	−0.1291	1.0191	0.4940	0.044*	
C8	−0.1500 (3)	0.95613 (18)	0.58534 (14)	0.0400 (6)	
H8	−0.2097	1.0140	0.6046	0.048*	
C9	−0.1111 (3)	0.8691 (2)	0.62345 (13)	0.0419 (6)	
H9	−0.1435	0.8687	0.6686	0.050*	
C10	−0.0255 (3)	0.78224 (18)	0.59682 (11)	0.0318 (5)	
C11	0.0132 (4)	0.6853 (2)	0.63601 (14)	0.0484 (7)	
H11A	0.1420	0.6738	0.6377	0.073*	
H11B	−0.0442	0.6251	0.6151	0.073*	
H11C	−0.0328	0.6936	0.6808	0.073*	
C12	0.1667 (3)	0.81024 (15)	0.83622 (10)	0.0239 (4)	
C13	−0.1090 (3)	0.75137 (18)	0.81651 (12)	0.0362 (5)	
H13	−0.1879	0.6982	0.8019	0.043*	
C14	−0.1789 (3)	0.84386 (17)	0.83828 (12)	0.0360 (5)	
H14	−0.3034	0.8565	0.8383	0.043*	
C15	−0.0596 (3)	0.91746 (18)	0.86010 (14)	0.0392 (6)	
H15	−0.1039	0.9820	0.8763	0.047*	
C16	0.4168 (3)	0.71305 (15)	0.79991 (10)	0.0254 (4)	
C17	0.4576 (3)	0.62233 (17)	0.83289 (13)	0.0337 (5)	
H17	0.4291	0.6148	0.8782	0.040*	
C18	0.5409 (3)	0.54212 (18)	0.79914 (15)	0.0417 (6)	
H18	0.5700	0.4789	0.8211	0.050*	
C19	0.5813 (3)	0.5547 (2)	0.73329 (15)	0.0445 (6)	
H19	0.6388	0.4999	0.7100	0.053*	
C20	0.5387 (3)	0.6461 (2)	0.70137 (13)	0.0433 (6)	
H20	0.5663	0.6530	0.6560	0.052*	
C21	0.4562 (3)	0.72886 (19)	0.73387 (12)	0.0328 (5)	
C22	0.4127 (4)	0.8309 (2)	0.70067 (14)	0.0513 (7)	
H22A	0.4773	0.8877	0.7225	0.077*	
H22B	0.2845	0.8439	0.7036	0.077*	
H22C	0.4481	0.8275	0.6543	0.077*	

### Atomic displacement parameters (Å^2^)

**Table:**

	*U*^11^	*U*^22^	*U*^33^	*U*^12^	*U*^13^	*U*^23^
O1	0.0202 (8)	0.0232 (7)	0.0447 (9)	−0.0012 (6)	−0.0002 (6)	−0.0071 (6)
O2	0.0207 (8)	0.0268 (7)	0.0389 (8)	0.0008 (6)	−0.0035 (6)	−0.0081 (6)
N1	0.0227 (9)	0.0239 (8)	0.0404 (10)	−0.0021 (7)	−0.0004 (8)	−0.0044 (8)
N2	0.0261 (10)	0.0239 (9)	0.0540 (12)	0.0003 (7)	−0.0015 (9)	−0.0096 (8)
N3	0.0246 (10)	0.0255 (9)	0.0433 (11)	−0.0027 (7)	0.0012 (8)	−0.0061 (8)
N4	0.0274 (11)	0.0249 (9)	0.0503 (11)	0.0003 (7)	−0.0039 (9)	−0.0087 (9)
C1	0.0213 (10)	0.0214 (9)	0.0292 (10)	−0.0003 (7)	−0.0012 (8)	0.0014 (8)
C2	0.0232 (12)	0.0315 (12)	0.0490 (14)	−0.0067 (9)	−0.0006 (10)	−0.0074 (11)
C3	0.0199 (11)	0.0410 (13)	0.0499 (13)	0.0042 (9)	−0.0008 (10)	−0.0085 (11)
C4	0.0296 (12)	0.0316 (12)	0.0567 (15)	0.0092 (9)	−0.0024 (11)	−0.0134 (11)
C5	0.0183 (10)	0.0241 (10)	0.0357 (11)	−0.0007 (7)	−0.0015 (8)	−0.0041 (8)
C6	0.0259 (11)	0.0275 (10)	0.0373 (11)	−0.0023 (8)	0.0013 (9)	−0.0001 (9)
C7	0.0269 (12)	0.0251 (10)	0.0587 (15)	−0.0006 (8)	−0.0047 (11)	−0.0002 (10)
C8	0.0259 (12)	0.0328 (12)	0.0614 (15)	0.0031 (9)	−0.0020 (11)	−0.0210 (11)
C9	0.0304 (13)	0.0549 (15)	0.0403 (13)	−0.0003 (11)	0.0010 (10)	−0.0152 (12)
C10	0.0232 (11)	0.0375 (12)	0.0346 (12)	−0.0014 (9)	−0.0031 (8)	−0.0004 (10)
C11	0.0465 (16)	0.0536 (16)	0.0450 (14)	0.0039 (12)	−0.0021 (12)	0.0111 (12)
C12	0.0229 (11)	0.0226 (9)	0.0260 (9)	−0.0012 (8)	−0.0022 (8)	0.0010 (8)
C13	0.0246 (12)	0.0349 (12)	0.0492 (13)	−0.0044 (9)	0.0013 (10)	−0.0113 (11)
C14	0.0226 (12)	0.0401 (12)	0.0453 (12)	0.0021 (9)	−0.0006 (10)	−0.0090 (11)
C15	0.0315 (13)	0.0321 (12)	0.0541 (14)	0.0064 (9)	−0.0028 (11)	−0.0128 (11)
C16	0.0175 (10)	0.0240 (10)	0.0346 (11)	−0.0008 (8)	−0.0011 (8)	−0.0047 (8)
C17	0.0271 (12)	0.0289 (11)	0.0452 (13)	−0.0003 (9)	0.0037 (10)	0.0030 (10)
C18	0.0302 (13)	0.0240 (11)	0.0709 (17)	0.0006 (9)	0.0035 (12)	−0.0010 (11)
C19	0.0294 (13)	0.0401 (13)	0.0640 (16)	0.0009 (10)	0.0028 (12)	−0.0244 (12)
C20	0.0332 (14)	0.0600 (17)	0.0368 (12)	0.0010 (12)	0.0037 (10)	−0.0129 (12)
C21	0.0255 (12)	0.0375 (12)	0.0353 (11)	−0.0013 (9)	−0.0024 (9)	−0.0001 (10)
C22	0.0511 (17)	0.0592 (18)	0.0435 (14)	0.0078 (14)	0.0015 (13)	0.0192 (13)

### Geometric parameters (Å, °)

**Table:**

O1—C1	1.362 (2)	C9—C10	1.394 (3)
O1—C5	1.408 (2)	C9—H9	0.9500
O2—C12	1.360 (2)	C10—C11	1.503 (4)
O2—C16	1.409 (2)	C11—H11A	0.9800
N1—C1	1.315 (3)	C11—H11B	0.9800
N1—C2	1.337 (3)	C11—H11C	0.9800
N2—C4	1.332 (3)	C13—C14	1.369 (3)
N2—C1	1.327 (2)	C13—H13	0.9500
N3—C12	1.317 (3)	C14—C15	1.375 (3)
N3—C13	1.343 (3)	C14—H14	0.9500
N4—C12	1.323 (3)	C15—H15	0.9500
N4—C15	1.339 (3)	C16—C17	1.375 (3)
C2—C3	1.378 (3)	C16—C21	1.389 (3)
C2—H2	0.9500	C17—C18	1.384 (3)
C3—C4	1.376 (3)	C17—H17	0.9500
C3—H3	0.9500	C18—C19	1.382 (4)
C4—H4	0.9500	C18—H18	0.9500
C5—C6	1.379 (3)	C19—C20	1.376 (4)
C5—C10	1.386 (3)	C19—H19	0.9500
C6—C7	1.387 (3)	C20—C21	1.394 (3)
C6—H6	0.9500	C20—H20	0.9500
C7—C8	1.377 (4)	C21—C22	1.506 (3)
C7—H7	0.9500	C22—H22A	0.9800
C8—C9	1.388 (4)	C22—H22B	0.9800
C8—H8	0.9500	C22—H22C	0.9800
			
C1—O1—C5	118.13 (15)	C10—C11—H11C	109.5
C12—O2—C16	117.89 (16)	H11A—C11—H11C	109.5
C1—N1—C2	114.70 (18)	H11B—C11—H11C	109.5
C4—N2—C1	114.36 (19)	N3—C12—N4	128.6 (2)
C12—N3—C13	114.84 (19)	N3—C12—O2	118.38 (18)
C12—N4—C15	114.59 (19)	N4—C12—O2	113.02 (17)
N1—C1—N2	128.94 (19)	N3—C13—C14	122.6 (2)
N1—C1—O1	118.79 (17)	N3—C13—H13	118.7
N2—C1—O1	112.27 (17)	C14—C13—H13	118.7
N1—C2—C3	122.7 (2)	C13—C14—C15	116.5 (2)
N1—C2—H2	118.6	C13—C14—H14	121.8
C3—C2—H2	118.6	C15—C14—H14	121.8
C2—C3—C4	116.2 (2)	N4—C15—C14	122.8 (2)
C2—C3—H3	121.9	N4—C15—H15	118.6
C4—C3—H3	121.9	C14—C15—H15	118.6
N2—C4—C3	123.1 (2)	C17—C16—C21	123.1 (2)
N2—C4—H4	118.4	C17—C16—O2	118.68 (19)
C3—C4—H4	118.4	C21—C16—O2	117.95 (19)
C6—C5—C10	123.2 (2)	C16—C17—C18	119.0 (2)
C6—C5—O1	118.85 (19)	C16—C17—H17	120.5
C10—C5—O1	117.69 (19)	C18—C17—H17	120.5
C5—C6—C7	119.1 (2)	C17—C18—C19	119.6 (2)
C5—C6—H6	120.4	C17—C18—H18	120.2
C7—C6—H6	120.4	C19—C18—H18	120.2
C8—C7—C6	119.4 (2)	C20—C19—C18	120.3 (2)
C8—C7—H7	120.3	C20—C19—H19	119.9
C6—C7—H7	120.3	C18—C19—H19	119.9
C7—C8—C9	120.5 (2)	C19—C20—C21	121.8 (2)
C7—C8—H8	119.7	C19—C20—H20	119.1
C9—C8—H8	119.7	C21—C20—H20	119.1
C8—C9—C10	121.4 (2)	C16—C21—C20	116.2 (2)
C8—C9—H9	119.3	C16—C21—C22	120.9 (2)
C10—C9—H9	119.3	C20—C21—C22	122.9 (2)
C5—C10—C9	116.4 (2)	C21—C22—H22A	109.5
C5—C10—C11	120.8 (2)	C21—C22—H22B	109.5
C9—C10—C11	122.8 (2)	H22A—C22—H22B	109.5
C10—C11—H11A	109.5	C21—C22—H22C	109.5
C10—C11—H11B	109.5	H22A—C22—H22C	109.5
H11A—C11—H11B	109.5	H22B—C22—H22C	109.5
			
C2—N1—C1—N2	0.2 (3)	C13—N3—C12—N4	1.1 (3)
C2—N1—C1—O1	−179.1 (2)	C13—N3—C12—O2	−179.6 (2)
C4—N2—C1—N1	0.5 (4)	C15—N4—C12—N3	−1.2 (3)
C4—N2—C1—O1	179.9 (2)	C15—N4—C12—O2	179.5 (2)
C5—O1—C1—N1	−8.8 (3)	C16—O2—C12—N3	13.5 (3)
C5—O1—C1—N2	171.72 (19)	C16—O2—C12—N4	−167.07 (18)
C1—N1—C2—C3	−0.5 (4)	C12—N3—C13—C14	0.3 (3)
N1—C2—C3—C4	0.1 (4)	N3—C13—C14—C15	−1.3 (4)
C1—N2—C4—C3	−0.9 (4)	C12—N4—C15—C14	0.0 (4)
C2—C3—C4—N2	0.7 (4)	C13—C14—C15—N4	1.1 (4)
C1—O1—C5—C6	92.2 (2)	C12—O2—C16—C17	−97.0 (2)
C1—O1—C5—C10	−93.2 (2)	C12—O2—C16—C21	88.6 (2)
C10—C5—C6—C7	−0.3 (3)	C21—C16—C17—C18	−0.4 (3)
O1—C5—C6—C7	174.07 (18)	O2—C16—C17—C18	−174.54 (19)
C5—C6—C7—C8	0.1 (3)	C16—C17—C18—C19	0.0 (3)
C6—C7—C8—C9	0.4 (3)	C17—C18—C19—C20	−0.2 (4)
C7—C8—C9—C10	−0.7 (4)	C18—C19—C20—C21	0.7 (4)
C6—C5—C10—C9	0.0 (3)	C17—C16—C21—C20	0.9 (3)
O1—C5—C10—C9	−174.42 (19)	O2—C16—C21—C20	175.07 (19)
C6—C5—C10—C11	178.4 (2)	C17—C16—C21—C22	−178.4 (2)
O1—C5—C10—C11	4.0 (3)	O2—C16—C21—C22	−4.2 (3)
C8—C9—C10—C5	0.5 (3)	C19—C20—C21—C16	−1.1 (4)
C8—C9—C10—C11	−177.9 (2)	C19—C20—C21—C22	178.2 (2)
